# Grape Seed Proanthocyanidins (GSPs) Inhibit the Growth of Cervical Cancer by Inducing Apoptosis Mediated by the Mitochondrial Pathway

**DOI:** 10.1371/journal.pone.0107045

**Published:** 2014-09-04

**Authors:** Qing Chen, Xiao-Fang Liu, Peng-Sheng Zheng

**Affiliations:** 1 The Department of Reproductive Medicine, The First Affiliated Hospital of Medical College, Xi’an Jiaotong University, Xi’an, China; 2 The Department of Biochemistry and Molecular Biology, Medical College of Xi’an Jiaotong University, Xi’an, China; 3 The Department of Pharmacology, Medical College of Xi’an Jiaotong University, Xi’an, China; 4 The Section of Cancer Research, Key Laboratory of Environment and Genes Related to Diseases, Ministry of Education of the People’s Republic of China, Xi’an, China; University of Toronto, Canada

## Abstract

Grape seed proanthocyanidins (GSPs), a biologically active component of grape seeds, have been reported to possess a wide array of pharmacological and biochemical properties. Recently, the inhibitory effects of GSPs on various cancers have been reported, but their effects on cervical cancer remain unclear. Here, we explored the effect of GSPs on cervical cancer using in vitro and in vivo models. In vitro, the treatment of HeLa and SiHa cells with GSPs resulted in a significant inhibition of cell viability. Further investigation indicated that GSPs led to the dose-dependent induction of apoptosis in cancer cells. The underlying mechanism was associated with increased expression of the pro-apoptotic protein Bak-1, decreased expression of the anti-apoptotic protein Bcl-2, the loss of mitochondrial membrane potential, and the activation of caspase-3, suggesting that GSPs induced cervical cancer cell apoptosis through the mitochondrial pathway. In addition, the administration of GSPs (0.1%, 0.2%, and 0.4%, w/v) as a supplement in drinking water significantly inhibited the tumor growth of HeLa and SiHa cells in athymic nude mice, and the number of apoptotic cells in those tumors was also increased significantly. Taken together, our studies demonstrated that GSPs could inhibit the growth of cervical cancer by inducing apoptosis through the mitochondrial pathway, which provides evidence indicating that GSPs may be a potential chemopreventive and/or chemotherapeutic agent for cervical cancer.

## Introduction

Cervical cancer is the third most common cancer [1] and the fourth leading cause of cancer-related death among women worldwide [2]. Approximately 80% of cervical cancer cases occur in developing countries, where approximately 529,000 new cases are detected every year, with nearly half of these patients dying [3]. In developing countries, due to the lack of screening and reduced access to appropriate therapeutic facilities and drugs, the incidence and mortality rates of cervical cancer rank second after breast cancer [4]. Many cases have developed into invasive cervical cancers at the time of diagnosis, and the patients are no longer candidates for radical surgical therapy. Although chemotherapy and radiotherapy are still the major treatments for invasive cervical cancer, the five-year survival rate is limited because of the limited efficacy and high toxicity of many anticancer drugs. Therefore, the exploration and development of more effective and less toxic therapeutic agents are required.

Epidemiologic studies have demonstrated that the consumption of a vegetable- and fruit-based diet significantly reduces the risk of cancer [5], [6], which offers promising new options for the development of more effective chemopreventive or chemotherapeutic strategies for various cancers. Recently, many phytochemicals of different chemical natures isolated from fruits and vegetables have been revealed to have potential chemopreventive and/or chemotherapeutic effects against cancers [7], [8], such as catechins, bioflavonoids, phyto-estrogens and proanthocyanidins [9], [10]. Grape seed proanthocyanidins (GSPs), a polyphenolic mixture, mainly contain 70%–95% proanthocyanidins, which constitute dimers, trimers, tetramers, and oligomers/polymers of monomeric catechins and/or (−)-epicatechins [11]–[13]. GSPs have been demonstrated to have minimal toxicity in vivo and effective anticancer effects on various human cancers [14], such as human prostate cancer [15], human colorectal cancer [16], [17], human non-small cell lung cancer [18], [19], pancreatic cancer [20], and head and neck squamous cancer [21]. Nevertheless, at present, no studies have examined the effects of GSPs on cervical cancer.

In this study, GSPs were found to be able to inhibit cervical cancer growth in vitro and in vivo, providing convincing evidence for the pharmacologic activity of GSPs against cervical cancer.

## Materials and Methods

### Antibodies, reagents and chemicals

Antibodies specific for Bcl-2, Bak-1 and β-actin were purchased from Santa Cruz Biotechnology, Inc. (Santa Cruz, CA). Secondary antibodies conjugated to horseradish peroxidase were purchased from Thermo Fisher Scientific, Inc. (New York, NY). The Annexin V-conjugated FITC apoptosis detection kit was obtained from BD Pharmaceuticals (Franklin Lakes, NJ). The JC-1 mitochondrial membrane potential detection kit and Caspase-3 activity detection kit were purchased from NanJing KeyGen Biotech Co., Ltd. (Nanjing, China), and the in situ cell death detection kit was purchased from Roche Diagnostic Corporation (Indianapolis, IN). MTT (3-(4,5-dimethyl-2-yl)-2,5-diphenyl tetrazolium bromide), PI (Propidium iodide) and DAPI (2-(4-amidinophenyl)-6-indolecarbamidine dihydrochloride) were obtained from Sigma Chemical Co. (St. Louis, MO). The GSPs were purchased from Jianfeng Natural Product R&D Co., Ltd. (Tianjin, China).

### Cell lines and cell culture

The human cervical cancer cell lines SiHa and HeLa were purchased from American Type Culture Collection (Manassas, VA). The cell lines were cultured as monolayers in Dulbecco’s modified Eagle’s medium (Sigma-Aldrich, St. Louis, USA) supplemented with 10% heat-inactivated fetal bovine serum (Invitrogen, Carlsbad, USA) at 37°C in 5% CO_2_. GSPs were dissolved in a small amount of dimethylsulfoxide (DMSO, 100 µl) prior to addition to the media. The maximum concentration of DMSO in the media did not exceed 0.1% (v/v), and cells treated with DMSO alone served as a vehicle control.

### MTT assay for cell viability

The effect of GSPs on cell viability was determined using an MTT assay as described previously [22]. Briefly, cells were plated in 96-well culture plates at 5×10^3^ cells per well and incubated overnight. Cells were treated with GSPs at various concentrations for 24, 48 or 72 h. At the end of the stimulation time, MTT was added to each well. The resulting formazan was then dissolved in 100 µL of dimethyl sulfoxide (DMSO), and the absorbance was recorded at 540 nm using a Bio-Rad 3350 microplate reader.

### Flow cytometric detection of apoptosis

The effects of GSPs on the apoptosis of cervical cancer cells were analyzed by flow cytometry using the Annexin V-conjugated FITC Apoptosis detection kit (BD, Franklin Lakes, NJ). Briefly, after treatment with GSPs for 48 h, cells were harvested, washed with PBS and incubated with Annexin V-FITC and PI for 10 min in the dark. Then, the stained cells were detected using a FACSCalibur flow cytometer (Becton Dickinson Franklin Lakes, NJ).

### Cell cycle analysis

Cells were treated with indicated concentrations of GSPs for 48 h, then harvested, washed with cold PBS, and followed by fixation with ice-cold 70% ethanol overnight at 4°C. After washed twice with PBS, the cells were stained with fluorescent probe solution containing 50 µg/ml PI and 1 mg/ml RNaseA on ice in the dark for 30 min. The cell cycle was analyzed by FACSCalibur flow cytometer (BD biosciences, San Jose, USA) using CellQuest software.

### Western blot analysis

Following treatment with GSPs, HeLa and SiHa cells were harvested, washed with cold PBS and lysed with ice-cold lysis buffer supplemented with protease inhibitors as described previously [23]. For western blot analysis, proteins were separated by SDS-PAGE and transferred onto PVDF membranes by wet transfer. After blocking with 5% fat-free milk, the membrane was incubated with the primary antibody at 4°C overnight. After washing, the membrane was incubated with the appropriate horseradish peroxidase-conjugated secondary antibody for 1 h, and the protein bands were visualized using enhanced chemiluminescence reagents (Millipore, Billerica, USA) on x-ray film.

### Assay for mitochondrial membrane potential

Changes in the mitochondrial membrane potential of cervical cancer cells treated with GSPs were measured by flow cytometry using the fluorescent lipophilic cationic probe JC-1 detection kit according to the manufacturer’s protocol. JC-1 accumulates selectively within normal mitochondria to form multimer J-aggregates that emit red fluorescence. JC-1 cannot aggregate in mitochondria with altered mitochondrial membrane potential and remains in the cytoplasm in monomeric form, fluorescing green. Thus, the color of the dye changes from orange to green, depending on the mitochondrial membrane potential, and can be analyzed by flow cytometry in the FITC channel.

### The detection of caspase-3 activity

The activity of caspase-3 was determined using the apo target kit according to the manufacturer’s protocol. Briefly, the cells were treated with GSPs for 48 h and then harvested. Cell lysates were prepared as described previously [23]. Samples of the cell lysates (150 µg protein per sample) were mixed with reaction buffer and substrate and incubated for 4 h at 37°C. The absorbance was then measured at 405 nm, and the sample readings were calculated by subtracting the absorbance of blank samples.

### Animals and tumor xenograft model

Female BALB/C nude mice (6–7 weeks old) were obtained from Slac Laboratory Animal Co., Ltd (Shanghai, China) and housed in the Animal Resource Facility at the Medical College of Xi’an Jiaotong University that was maintained at a constant temperature (22°C–25°C) and humidity (40–50%). All animals had free access to drinking water and food, and received humane treatment in accordance with the internationally accepted principles for laboratory animal use and care (European Community Guidelines, EEC Directive of 1986; 86/609/EEC). The animal protocol used in this study was approved by the Animal Care and Use Committee of the Medical College of Xi’an Jiaotong University.

To determine the efficacy of GSPs against cervical cancer growth in vivo, two tumor xenograft models were used. Model 1: Mice were randomly divided into four groups with six mice per group, Group 1, the control; Group 2, GSPs (0.1%, w/v); Group 3, GSPs (0.2%, w/v); Group 4, GSPs (0.4%, w/v), and the mice received GSPs for at least 10 days before the implantation of tumor cells. Model 2: Mice were randomly divided into two groups with six mice per group, Group 1, the control; Group 2, GSPs (0.4%, w/v), and the mice began to receive GSPs on the 12th day after the implantation of tumor cells. Exponentially growing HeLa and SiHa cells (2×10^6^ in 100 µl PBS) were injected subcutaneously in the right flank of each mouse, respectively. GSPs was dissolved in the distilled water, and the mice received GSPs by drinking water freely. The control mice received the distilled water. The tumor growth was monitored regularly using vernier calipers throughout the experiment, and the volumes of tumors were calculated using the following formula: Volume = (length×width^2^)/2. At the termination of the experiment, the mice were sacrificed by decapitation under 25% urethane anaesthesia, and the tumor mass was harvested and weighed. A portion of the tumor tissue was paraffin-embedded and the other portion was frozen in liquid nitrogen for the further analysis.

### Histopathological examination

The paraffin sections of tumor xenografts were deparaffinized in xylene and rehydrated through descending concentrations of ethanol according to routine methods and were stained with hematoxylin and eosin (H&E) as previously described [24]. All sections were examined under a light microscope.

### TUNEL assay for apoptosis of cancer cells

The TUNEL assay was performed using an in situ cell death detection kit (Roche Corporation, USA) following the manufacturer’s protocol. Briefly, after dewaxing and rehydrating the tumor sections, they were incubated with proteinase K at 37°C for 15 min. The permeabilized sections were incubated with TUNEL reaction mixture at 37°C for 60 min in the dark. After being rinsed 3 times with PBS, the sections were incubated with converter-POD at 37°C for 30 min, followed by DAB substrate development. The nuclei were counterstained with hematoxylin, and TUNEL-positive cells were examined and counted under a microscope.

### Fluorescence microscopy examination

Apoptotic nuclear morphology was assessed by staining cells with the fluorescent DNA-binding dye DAPI. After treatment of cervical cancer cells with GSPs for 48 h, the cells were stained with DAPI (0.5 mg/ml) for 5 min in the dark, and then, the nuclear morphology was observed under a fluorescence microscope (Olympus, Japan).

### Statistical analysis

All results were confirmed in three independent experiments. The data are expressed as means ± SD. Statistical comparisons were made by either simple one-way ANOVA followed by Tukey’s *post hoc* test or the Chi-Square test, and *p*<0.05 was considered statistically significant.

## Results

### GSPs inhibited the viability of cervical cancer cells in vitro

**Figure 1:**
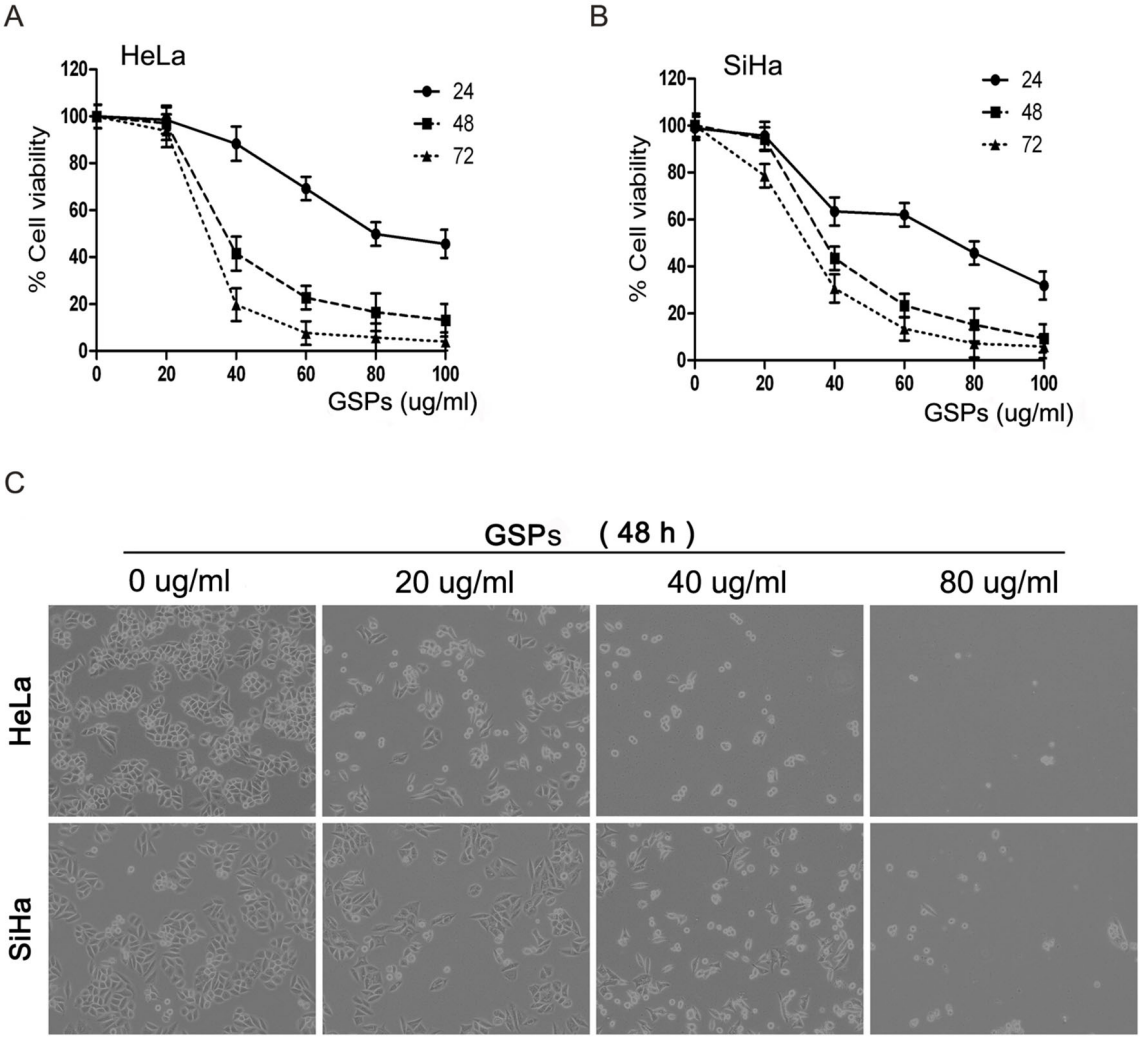
GSPs inhibited the proliferation of cervical cancer cells. (A) HeLa and (B) SiHa cells were treated with varying doses of GSPs, and the relative viability of the cells was assessed by MTT assay at 24 h, 48 h and 72 h. The results were expressed in terms of the percentage of control cells as the mean ± SD obtained from 3 separated experiments. (C) Growth inhibition and morphologic changes of HeLa and SiHa cells treated with GSPs for 48 h compared with control cells (non-GSPs-treated). Cells were photographed with inverted contrast microscopy (magnification, 40×).

The effect of GSPs on the viability of cervical cancer cells was first assessed using an MTT assay. The cervical cancer cell lines, including HeLa and SiHa cells, were treated with different doses of GSPs (0, 20, 40, 60, 80 and 100 µg/ml) for 24, 48 or 72 h. As shown in Fig. 1A, the treatment of HeLa cells with GSPs resulted in a significant reduction in the viability of the cells in a dose-dependent manner (*p*<0.05). In particular, a significant inhibitory effect was observed at 48 h after GSP treatment (5–85% reduction, *p*<0.05). Moreover, the effect was also time dependent (60 µg/ml GSPs, 25–90% reduction, *p*<0.05). Similar inhibitory effects were observed on SiHa cells treated with GSPs (Fig. 1B). All these results indicated that GSPs could effectively inhibit the viability of cervical cancer cells.

In addition, the cell viability of SiHa and HeLa cells following GSPs treatment was also observed under a phase-contrast microscope. As shown in Fig. 1C, after 48 hours of GSPs treatment, the density of both cell lines was significantly decreased in a dose-dependent manner. GSPs treatment also remarkably altered the morphology of the cervical cancer cells. After GSPs treatment, the HeLa and SiHa cells decreased in size, retracted from their neighbors, lost their flat and polygonal shape, and ultimately detached from the culture dish, suggesting that GSPs inhibited cervical cancer cell viability by inducing cell death.

### GSPs induced cell cycle arrest in cervical cancer cells

**Figure 2:**
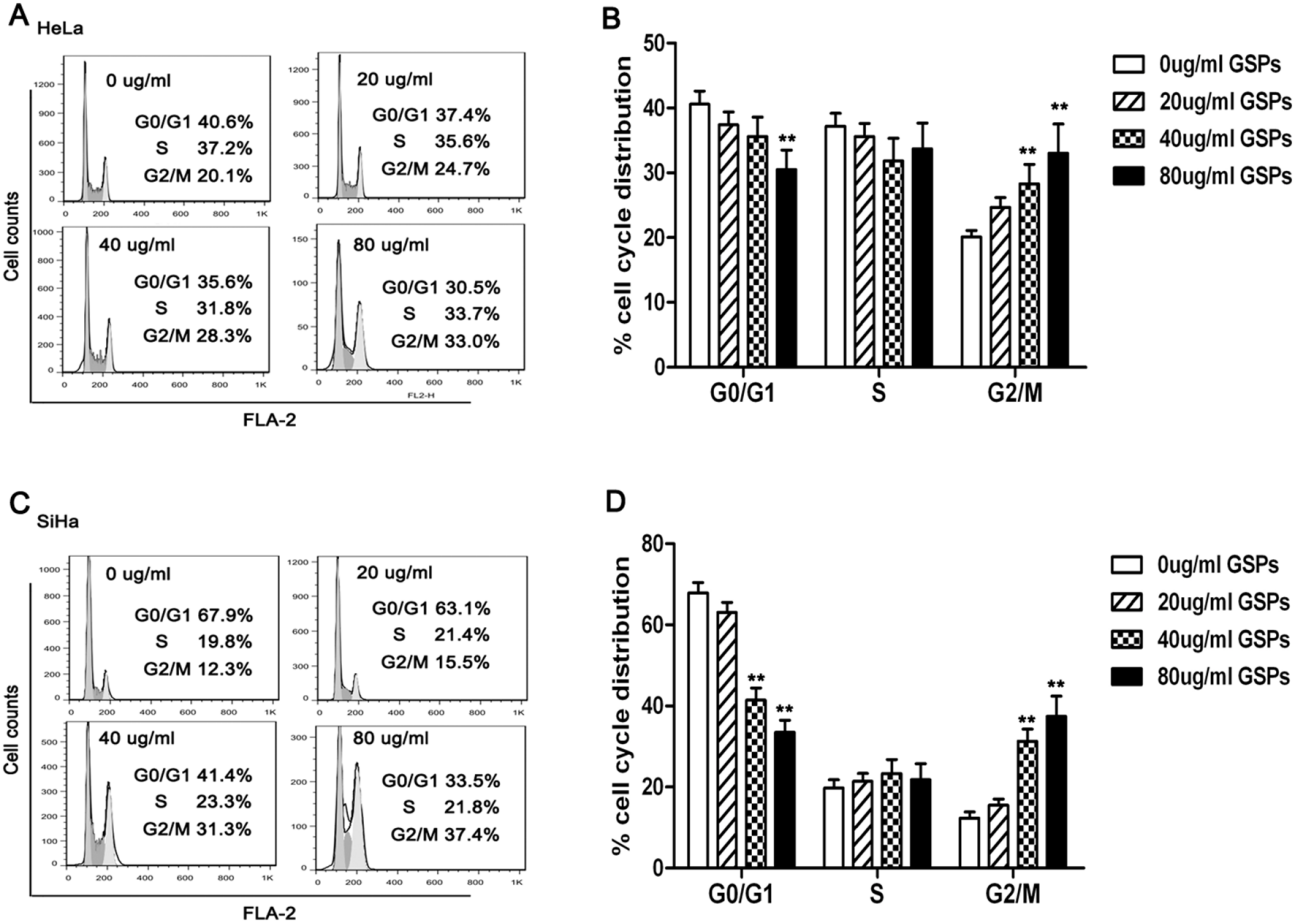
GSPs induced cell cycle arrest of cervical cancer cells. HeLa and SiHa cells were treated with various dose of GSPs for 48 h, and harvested. The cell cycle distribution was analyzed by flow cytometry. (A, C) The representative cell cycle histograms of HeLa and SiHa. (B) The treatment of HeLa cells with GSPs resulted in the accumulation of cells in G_2_/M phase. (D) The treatment of SiHa cells with GSPs led to the accumulation of cells in G_2_/M phase. Results were expressed as mean ± SD, *p<0.05 *vs.* control; **p<0.01 *vs.* control.

To clear whether the cervical cancer cell viability inhibition of GSPs is involved in the alteration in cell cycle progression, the cell cycle analysis was performed. HeLa and SiHa cells were treated with various dose of GSPs for 48 h, the distribution of cells in different phases of the cell cycle were analyzed by flow cytometer. As shown in Fig. 2A and B, the treatment of HeLa cells with GSPs resulted in a significant accumulation of cells in G2/M phase at higher concentrations (40 µg/ml and 80 µg/ml, *p*<0.01). Similar to HeLa cell results, a significant arrest of the cells in the G2/M phase of cell cycle was also observed in SiHa cells at 40 and 80 µg/ml concentration of GSPs (Fig. 2C and D, *p*<0.01). Together, these findings suggested that GSPs could induce the arrest of cell cycle in G_2_/M phase, which might be associated with the inhibitory effect of GSPs on cervical cancer cell.

### GSPs induced the apoptosis of cervical cancer cells

**Figure 3:**
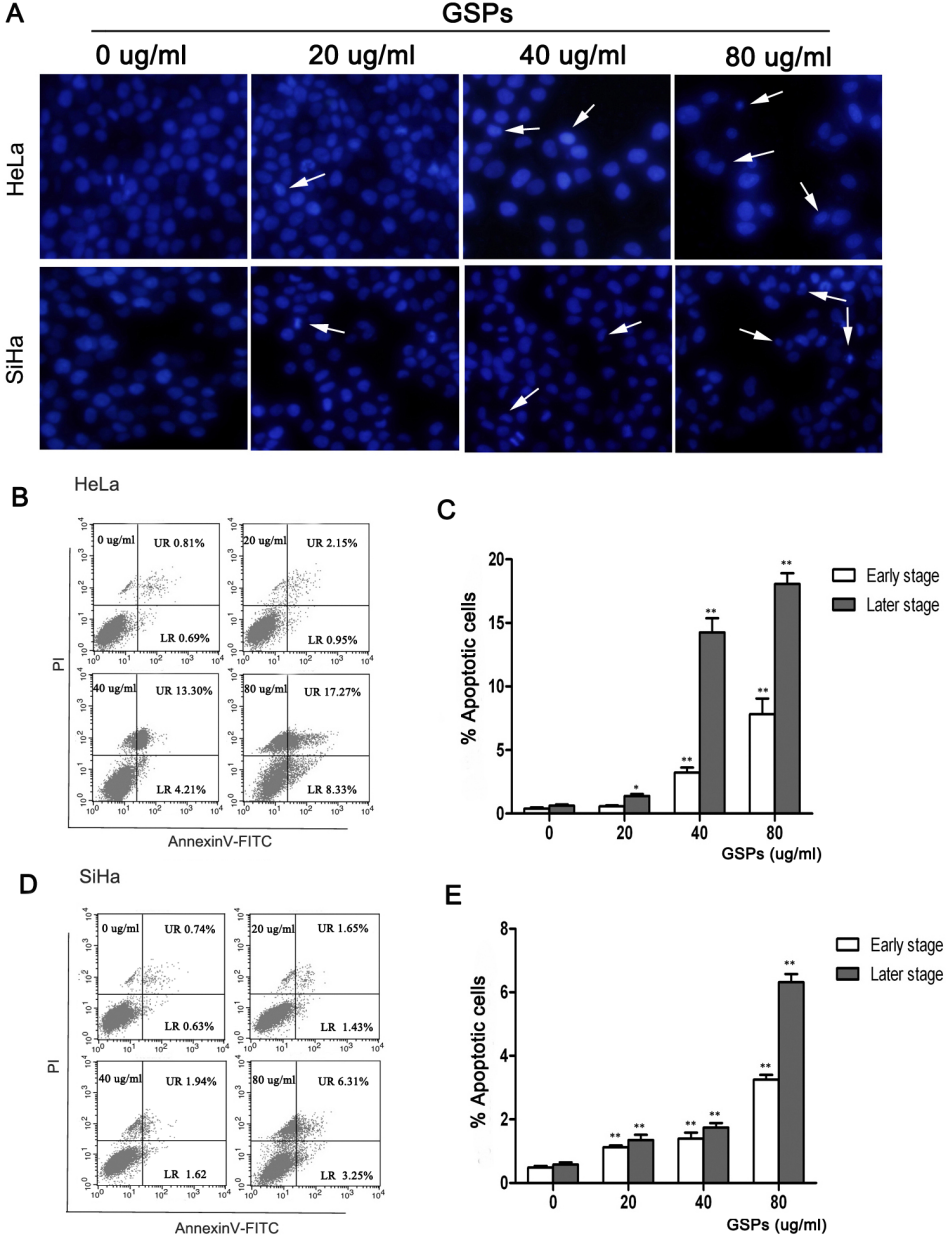
GSPs induced the apoptosis of cervical cancer cells in vitro. HeLa and SiHa cells were treated with varying doses of GSPs for 48 h and then harvested for analysis of apoptosis. (A) The morphological changes of nuclei were examined by fluorescence microscopy using DAPI staining. The arrow indicates nuclear condensation and an apoptotic body (magnification, 40×). Flow cytometry analysis of Annexin V-FITC/PI double-stained HeLa (B) and SiHa (C) cells. The low right (LR) quadrant of the histograms indicates the early apoptotic cells, and the upper right (UR) quadrant indicates the late apoptotic cells. The treatment of HeLa (B) and SiHa (C) cells with GSPs results in significant increases in the percentages of apoptotic cells (including early stage and late stage). Values are expressed as the mean ± SD of three experiments in duplicate, *p<0.05 *vs.* control; **p<0.01 *vs.* control.

To determine whether GSPs induced the apoptosis of cervical cancer cells, we observed the morphological change of cell nucleus using DAPI staining (Fig. 3A). A number of HeLa and SiHa cells treated with various doses of GSPs for 48 h were found to display classic apoptotic changes, such as chromatin condensation, karyopyknosis and apoptotic body formation. However, the untreated control SiHa and HeLa cells seldom displayed these features, suggesting that GSP treatment may induce the apoptosis of cervical cancer cells.

For further quantitative analysis of the apoptosis induced by GSPs, cells treated with various doses of GSPs were analyzed by flow cytometry using Annexin V-FITC/PI double staining. Apoptotic cells could be typed into early-stage apoptosis (Annexin V^+^ and PI^−^) and late-stage apoptosis (Annexin V^+^ and PI^+^), which are shown, respectively, in the lower right (LR) and upper right (UR) quadrants of the FACS histograms (Fig. 3B and D, left panel). The apoptosis results (including early-stage and late-stage) in both cell lines are further summarized in Figure 3 C and E. The percentage of total apoptotic cells in HeLa cells was 1.5% in control cells (early-stage: 0.69% and late-stage: 0.81%), 3.1% in cells treated with 20 µg/ml GSPs (early-stage: 0.95% and late-stage: 2.15%), 17.51% in cells treated with 40 µg/ml (early-stage: 4.21% and late-stage: 13.30%) and 25.6% in cells treated with 80 µg/ml (early-stage: 8.33% and late-stage: 17.27%). Similarly, those in SiHa cells were 1.37% in control cells (early-stage: 0.63% and late-stage: 0.74%), 3.08% in cells treated with 20 µg/ml GSPs (early-stage: 1.43% and late-stage: 1.65%), 3.56% in cells treated with 40 µg/ml (early-stage: 1.62% and late-stage: 1.94%) and 9.56% in cells treated with 80 µg/ml (early-stage: 3.25% and late-stage: 6.31%). All these results indicated that GSPs significantly induced the apoptosis (including early and late-stage apoptosis, *p*<0.01) of cervical cancer cells.

### GSPs induced the apoptosis of cervical cancer cells through the down-regulation of Bcl-2 and the up-regulation of Bak-1

**Figure 4:**
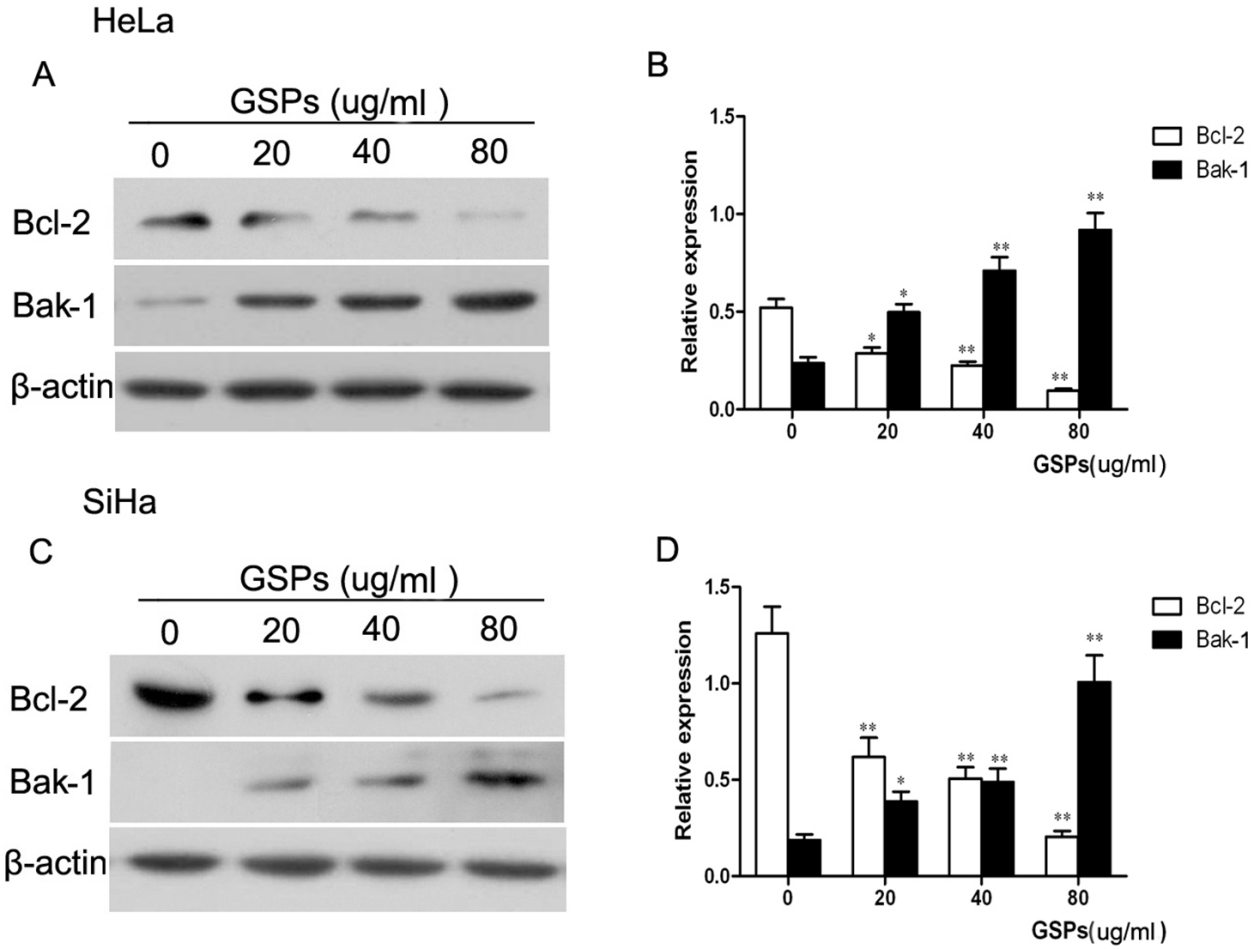
The effect of GSPs on the expression of Bcl-2 and Bak-1 in cervical cancer cells. The cells were treated with varying doses of GSPs for 48 h and then harvested. Cell lysates were prepared and subjected to western blot analysis. (A) Treatment of HeLa cells with GSPs resulted in a dose-dependent reduction of Bcl-2 expression and an increase in Bak-1 expression. (B) The relative expression of Bcl-2 and Bak-1 proteins in HeLa cells were calculated based on β-actin expression, which was used as loading control. (C) GSPs significantly decreased the expression of Bcl-2 and increased the expression of Bak-1 in SiHa cells. (D) The relative of expression levels of Bcl-2 and Bak-1 in SiHa cells are summarized. Representative blots are shown from three independent experiments, and values are expressed as the mean ± SD. *p<0.05 *vs.* control; **p<0.01 *vs.* control.

Both the Bcl-2 and Bak-1 proteins play crucial roles in the regulation of apoptosis [25], [26]. Thus, the expression of Bcl-2 and Bak-1 was examined by western blotting to determine whether these two proteins are involved in the induction of cervical cancer cell apoptosis by GSPs. The representative blots for HeLa and SiHa cells are shown in Fig. 4A and C, respectively, and the relative expression of these proteins was further calculated through normalization to β-actin expression and is summarized in Figure 4 B and D, respectively. The results showed that the expression of Bcl-2 in HeLa cells treated with GSPs for 48 hours was significantly decreased in a dose-dependent manner (Fig. 4B, *p*<0.05). Conversely, the expression of Bak-1 was significantly increased (Fig. 4B, *p*<0.05). Similar results were also found with SiHa cells (Fig. 4D, *p*<0.05). Therefore, GSPs induced the apoptosis of cervical cancer cells through the down-regulation of Bcl-2 and the up-regulation of Bak-1.

### GSPs induced the loss of mitochondrial membrane potential in cervical cancer cells and activated Caspase-3 protein

**Figure 5:**
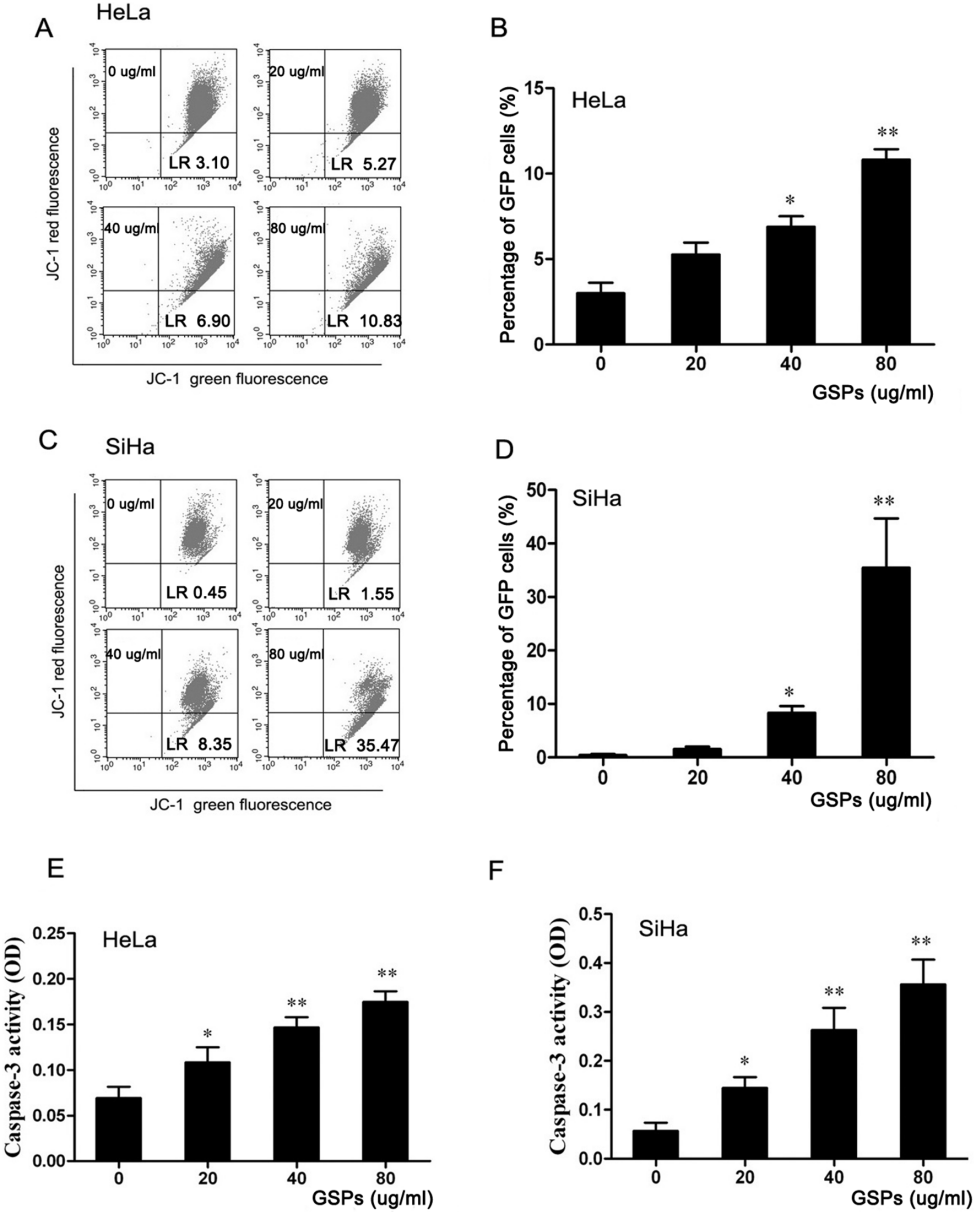
The apoptosis of cervical cancer cells induced by GSPs was mediated by the mitochondrial pathway. HeLa (A) and SiHa (C) cells were treated with the indicated doses of GSPs for 48 h and then harvested, stained with JC-1 dye, and finally analyzed by flow cytometry. GSPs resulted in a significant loss of mitochondrial membrane potential in HeLa (B) and SiHa cells (D). Caspase-3 activity in HeLa and SiHa cells was measured using a colorimetric protein assay, and treatment of HeLa (E) and SiHa (F) cells with GSPs resulted in a dose-dependent increase in the activity of caspase-3. GFP: Green fluorescence positive. Data are presented as the mean ± SD from three independent experiments, *p<0.05 *vs.* control; **p<0.01 *vs.* control.

It is well known that the intracellular translocation of Bcl-2 and Bak-1 can induce the loss of mitochondrial membrane potential, which is linked to the initiation and activation of the apoptotic process in cells [27], [28]. To explore the effect of GSPs on mitochondrial membrane potential, the integrity of the mitochondrial membrane of cells was determined by staining with JC-1, a cationic lipophilic dye. As shown in Fig. 5A and C, HeLa and SiHa cells treated with GSPs for 48 h were analyzed by FACS. The average percentage of green fluorescence-positive HeLa cells was 3.10% at 0.00 µg/ml, 5.27% at 20 µg/ml, 6.90% at 40 µg/ml and 10.83% at 80 µg/ml (Fig. 5B), while that of the SiHa cells was 0.45% at 0.00 µg/ml, 1.55% at 20 µg/ml, 8.35% at 40 µg/ml and 35.47% at 80 µg/ml (Fig. 5D). These results showed a significant increase in green fluorescence-positive cells with increasing doses of GSPs (*p*<0.01), suggesting that GSPs can induce cervical cancer cells to lose mitochondrial membrane potential. Therefore, GSPs induced the apoptosis of cervical cancer cells through the disruption of mitochondrial membrane potential.

Due to the loss of mitochondrial membrane potential, cytochrome c is released into the cytosol, which activates procaspase 9 in the apoptosome and leads to the cleavage of caspase-3 [29]. Subsequently, active cleaved caspase-3 can cleave a broad spectrum of target proteins and finally result in apoptotic cell death. To examine whether the apoptosis induced by GSPs is involved in cascade activation, the activities of caspase-3 were determined by a colorimetric assay. The results showed that the treatment of both HeLa and SiHa cells with GSPs for 48 h resulted in significant increase in caspase-3 activity in a dose-dependent manner (Fig. 5E and F), suggesting that the apoptosis of cervical cancer cells induced by GSPs occurred through the activation of the caspase-3 pathway.

### GSPs inhibited the initiation and progression of cervical cancer in vivo

Because GSPs could induce the apoptosis of cervical cancer cells in vitro, it was necessary to test whether GSPs could inhibit the initiation and tumor growth of cervical cancer cells in vivo. To test the effects of GSPs on the initiation and progression of human cervical cancer cells, nude mice were fed GSPs for 10 days before inoculation with cervical cancer cells. The growth of tumors was monitored in terms of tumor volume every three days. At the termination of the experiment, the tumors were excised after the mice were sacrificed, and the wet weights of the tumors were determined.

**Figure 6:**
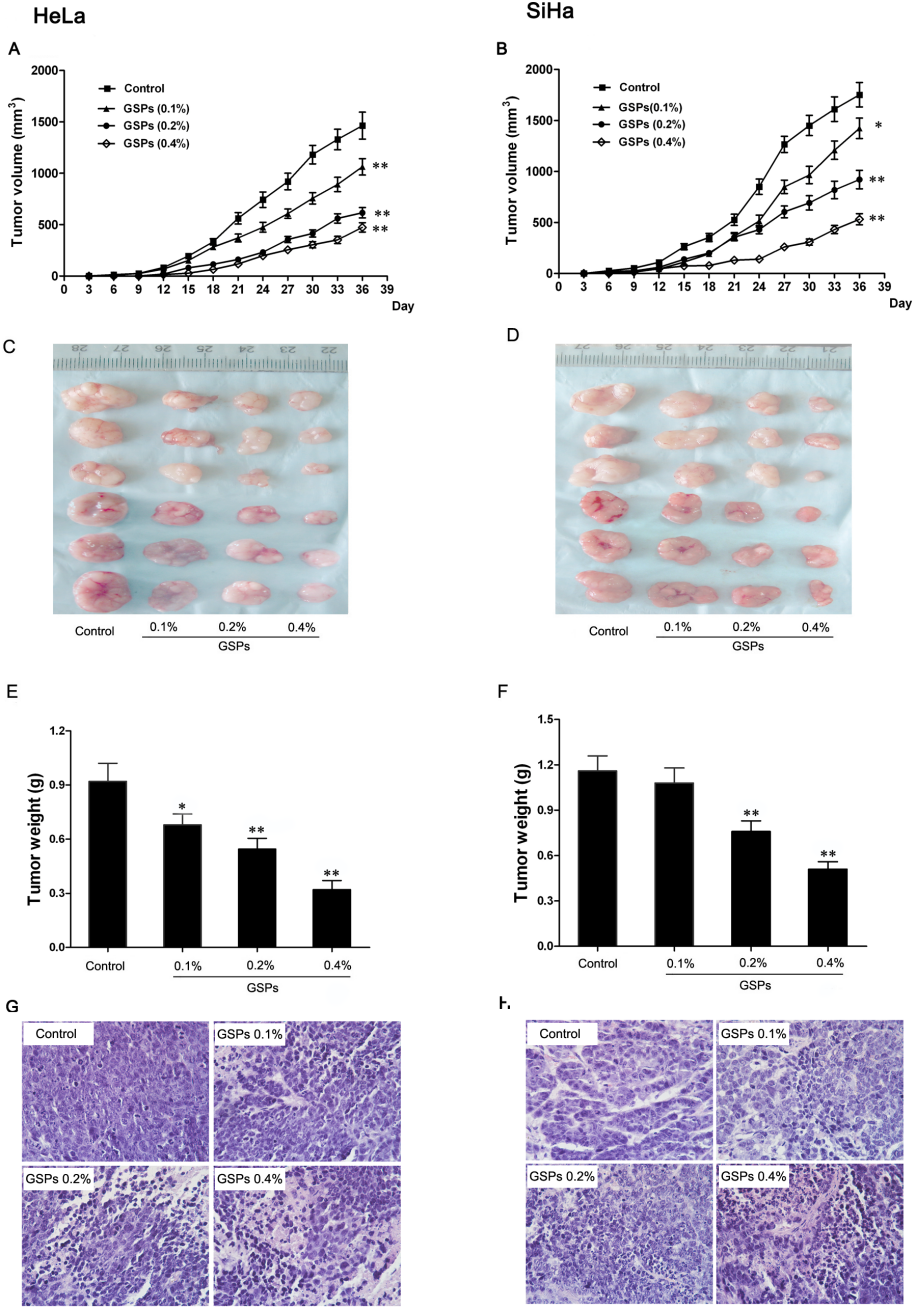
GSPs inhibited the growth of cervical cancer xenografts in vivo. Mice were *s.c.* inoculated in the right flank with 2×10^6^ tumor cells. (A and B) Average tumor volumes in each group were measured on a regular basis. At the termination of the experiment, the tumor mass was harvested (C and D), and the wet weight of the tumor was recorded (E and F). (G and H) The histopathological examination of tumor xenografts was performed under light microscope, (magnification, 40×). Values are expressed as the mean ± SD, and the statistical significance of differences was analyzed by one-way ANOVA followed by the Tukey test. *p<0.05 *vs.* control; **p<0.01 *vs.* control.

First, we found that tumor formation by HeLa cells in the control group occurred at 12 days after inoculation; however, tumor formation in the GSPs treatment group (GSPs, 0.2% and 0.4%) occurred approximately at 15 days after inoculation. Similar results were observed in SiHa cells, suggesting that GSPs inhibited the initiation of cervical cancer. Second, the growth rate of HeLa and SiHa tumor xenografts in mice treated with GSPs was significantly slower than that of the controls (Fig. 6A and B), which suggested that GSPs inhibited the growth of cervical cancer xenografts in nude mice. Ultimately, the average wet weight of tumor xenografts formed by HeLa cells following GSPs treatment was significantly less than that of the control (*p*<0.01, Fig. 6C and E). Similarly, the administration of GSPs also resulted in significant reduction in the wet weight of SiHa tumor xenografts (*p*<0.01, Fig. 6D and F). Additionally, the histopathological examination of tumor xenografts were performed under light microscope. As shown in Fig. 6G and H, obvious morphological changes were observed in the GSPs treated groups. Compared to the control group, the tumor tissues of GSPs treated groups, especially 0.4% GSPs group, presented widespread karyopyknosis, apoptotic body formation and cell disruption, suggesting that GSPs led to the apoptotic and necrotic death of cervical cancer cells. Collectively, these results suggested that GSPs could inhibit the initiation and growth of cervical cancer xenografts, exhibiting a chemopreventive effect against cervical cancer.

### Chemotherapeutic effect of GSPs on cervical cancer xenografts

**Figure 7:**
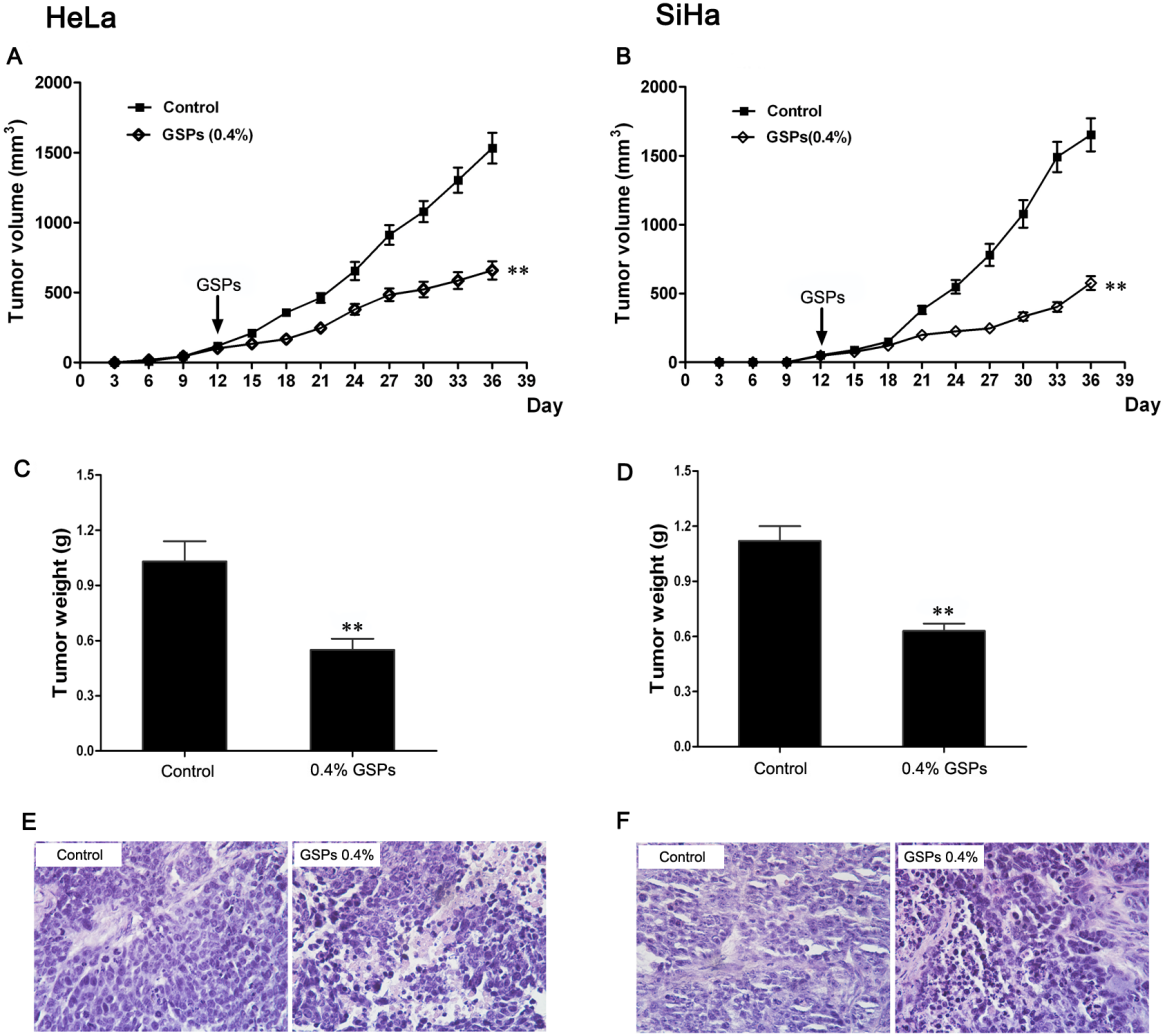
Chemotherapeutic effect of GSPs on cervical cancer cells grown as tumor xenografts. In the GSP-treated group, the mice were administered GSPs (0.4%) on the 12^th^ day after the implantation of tumor cells. (A and B) Tumor volumes were recorded every 3 days, and the administration of GSPs inhibited the growth of HeLa (A) and SiHa (B) tumor xenografts. (C and D) The tumor mass was harvested at the end of the experiment, and the wet weight of the tumor was measured. The administration of GSPs led to a significant reduction of the wet tumor weight. (E and F) The obvious morphological change of apoptotic cell death was observed in the tumor tissue of GSPs-treated group, but not in control group, (magnification, 40×). Values are expressed as the mean ± SD, **p<0.01 *vs.* control.

To evaluate the therapeutic effect of GSPs on the established tumors, mice were treated with GSPs (0.4%, w/v) on the 12^th^ day after implantation, after tumors had already formed. The growth of tumors was monitored regularly, and we found that treatment with GSPs lead to a significant reduction in the tumor growth rate. As shown in Fig. 7A and B, the tumor volumes of GSPs-treated mice were markedly reduced compared with control mice, suggesting that GSPs could inhibit the growth of HeLa and SiHa tumor xenografts. Moreover, at the termination of the experiment, the measurement of tumor wet weight revealed that the wet weight of tumors in the GSPs-treated group was significantly decreased compared with the control group (Fig. 7C and D, *p*<0.01), and the histopathological examination also found that the morphological change of tumor xenografts in the GSPs-treated group was also remarkable compared with the control group (Fig. 7E and F). Therefore, these results further demonstrated the inhibitory effect of GSPs against cervical cancer progression.

### GSPs induced cell apoptosis of cervical cancer xenografts in vivo

**Figure 8:**
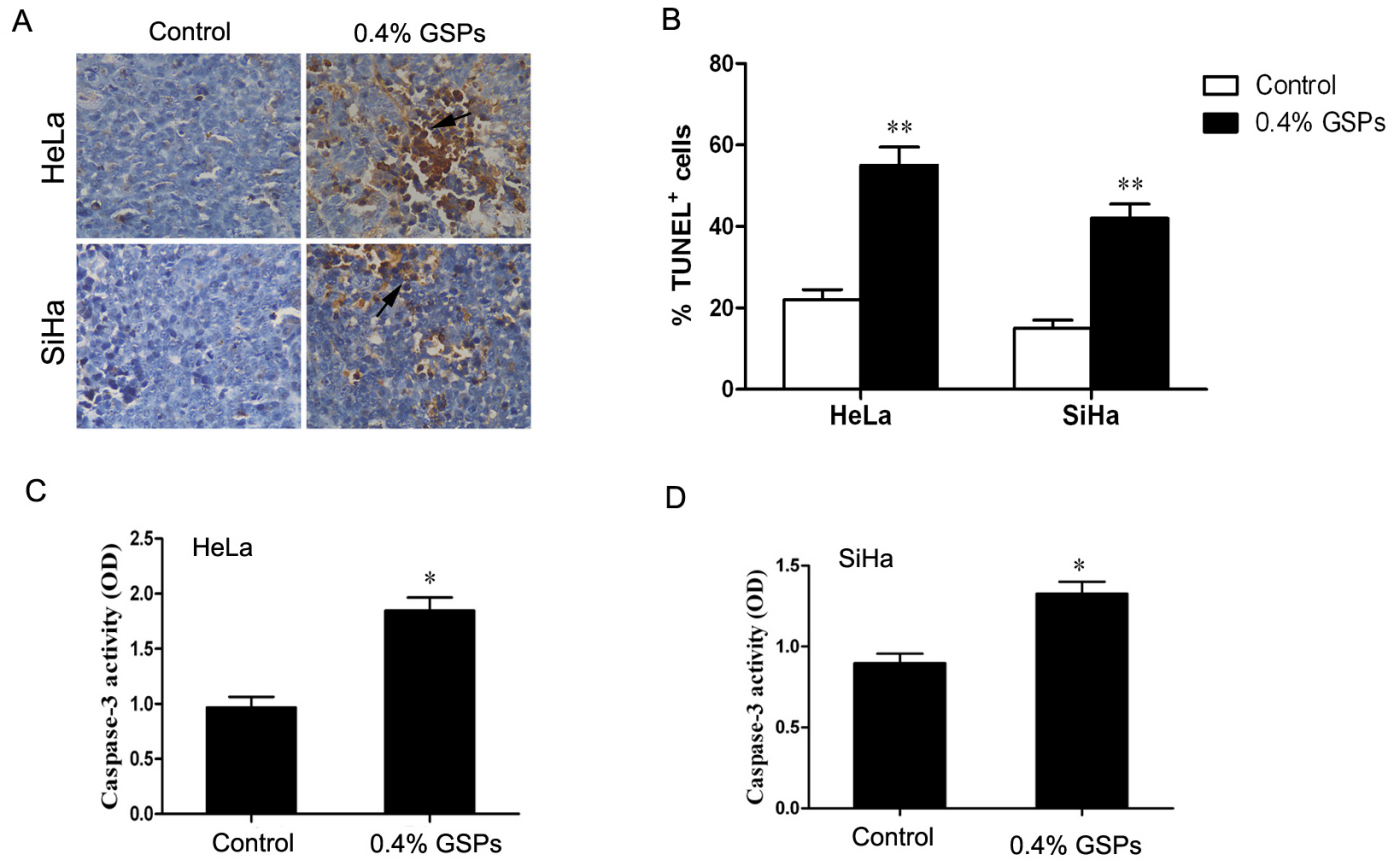
GSPs induced apoptotic cell death in cervical cancer xenografts. (A) Apoptotic cell death in HeLa and SiHa tumor xenografts was measured by TUNEL assay, and representative micrographs are shown (magnification, 40×). (B) Quantitative analysis of apoptosis in xenograft samples. The activities of caspase-3 in HeLa (C) and SiHa (D) tumor xenografts were determined using a colorimetric protein assay. Values are presented as mean ± SD, *p<0.05 *vs.* control; **p<0.01 *vs.* control.

Resistance to apoptosis is one of the important characteristic features of malignant tumors. To determine whether GSPs inhibit cervical cancer progression by inducing the apoptotic cell death of tumor cells, we evaluated apoptosis using the TUNEL assay on tumor tissues formed by SiHa and HeLa cells with or without GSPs treatment in model 1. The assay results are shown in Fig. 8A and are quantitatively summarized in Fig. 8B. The percentage of TUNEL-positive cells in tumor tissues from HeLa cells treated with 0.4% GSPs was approximately threefold higher than that of the control, and that of SiHa cells treated with 0.4% GSPs was fourfold higher than that of the control. Furthermore, the activity of caspase-3 was also detected to assess apoptotic effect of GSPs on cancer tissues formed by SiHa and HeLa cells. As shown in Fig. 8C and D, the activity of caspase-3 in HeLa xenografts was 1.85 in the 0.4% GSP group and 0.96 in the control group (*p*<0.05). The activity of caspase-3 in SiHa xenografts was 1.37 in the 0.4% GSPs group and 0.83 in the control group (*p*<0.05). Taken together, all these results indicated that GSPs induced apoptosis in vivo when GSPs were used to inhibit cervical cancer progression.

## Discussion

Although chemotherapy is still a fundamental option for the treatment of the majority of human invasive malignancies, its efficacy is mainly limited due to drug side effects and the rapid development of drug resistance. Therefore, it is still necessary to explore more effective anticancer compounds, which have less toxicity. Recently, GSPs, as a dietary botanical supplement, have been shown to have anticancer activity in some studies [15], [20], [30]–[32]. However, the effects of GSPs on cervical cancer currently remain unclear. We studied the effects of GSPs on cervical cancer for the first time using in vitro and in vivo models, and the results showed that GSPs have both chemopreventive and therapeutic efficacy against cervical cancer.

First, using the MTT assay, we found that the treatment of HeLa and SiHa cells with GSPs resulted in a dose-dependent reduction in cell viability (Fig. 1A and B). Second, cell shrinkage and detached cells were frequently observed in the GSPs-treated cells by phase-contrast microscopy (Fig. 1C), suggesting that GSPs significantly inhibited the proliferation of cervical cancer cells. It is well known that carcinogenesis is closely associated with uncontrolled cell cycle. Through cell cycle analysis, we found that GSPs led to a significant arrest of the cells in G2/M phase of cell cycle (Fig. 2). However, the growth arrest of cancer cells in G2/M phase provides an opportunity for cells to either undergo repair mechanisms or apoptosis. Interestingly, morphological changes of cell nucleus that are characteristic of apoptosis, such as condensation of nuclear chromatin and formation of apoptotic bodies, were observed under fluorescence microscopy in the GSPs-treated HeLa and SiHa cells stained by DAPI (Fig. 3A). Furthermore, flow cytometry analysis using Annexin V-FITC/PI staining showed that GSPs led to the apoptosis of HeLa and SiHa cells in a dose-dependent manner. Collectively, these data indicated that GSPs inhibited the viability of cervical cancer cells by inducing apoptosis in vitro. In addition, our study using tumor xenograft experiments indicated that GSPs also induced the apoptosis of cervical cancer cells in vivo (Fig. 8). Consistently, GSPs induced apoptosis in colorectal carcinoma, pancreatic cancer and breast carcinoma in vitro and in vivo [16], [20], [30], [33].

The induction of apoptosis was associated with the down-regulation of anti-apoptotic proteins and/or up-regulation of pro-apoptotic proteins. Bcl-2, as an important apoptosis-inhibiting protein, and Bak-1, an apoptosis-promoting protein, play key roles in the apoptotic process [28]. In this study, the GSPs-treated HeLa and SiHa cells were found to express significantly less Bcl-2 protein and more Bak-1 protein than the untreated cells (Fig. 4), suggesting that both Bcl-2 and Bak-1 play roles in GSPs-induced apoptosis in cervical cancer cells. It was reported that the activation of Bak-1 could disrupt the normal function of Bcl-2 and lead to the loss of mitochondrial membrane potential and enhancement of mitochondrial membrane permeability [34], [35], which in turn result in the release of Cytochrome c and apoptosis-inducing factors from mitochondria into the cytosol. Subsequently, cytosolic Cytochrome c, Apaf-1 and ATP form the apoptosome complex, which activates pro-caspase-9, finally leading to the activation of caspase-3, one of the key mediators of apoptosis, followed by cell death [36]. In the present study, the treatment of HeLa and SiHa cells with GSPs resulted in a significant loss of mitochondrial membrane potential and increased activity of caspase-3 (Fig. 5). Moreover, TUNEL assays and caspase-3 detection revealed that the tumor xenograft tissues from the GSPs-treated group contained more apoptotic cells and greater caspase-3 activity than the control (Fig. 8). All these results suggested that GSPs induced the apoptosis of cervical cancer cells through the mitochondrial pathway.

To determine whether GSPs also exert an inhibitory effect on cervical cancer in vivo, tumor xenograft experiments with HeLa and SiHa cells were performed on nude mice fed GSPs-containing water. The results showed that GSPs administration remarkably inhibited the initiation and progression of tumor xenografts (Fig. 6). Additionally, the GSPs treatment also inhibited tumor growth even if the tumor xenografts had been established (Fig. 7), suggesting that GSPs have the potential to be used not only as a preventive drug but also as a therapeutic treatment for cervical cancer.

**Figure 9:**
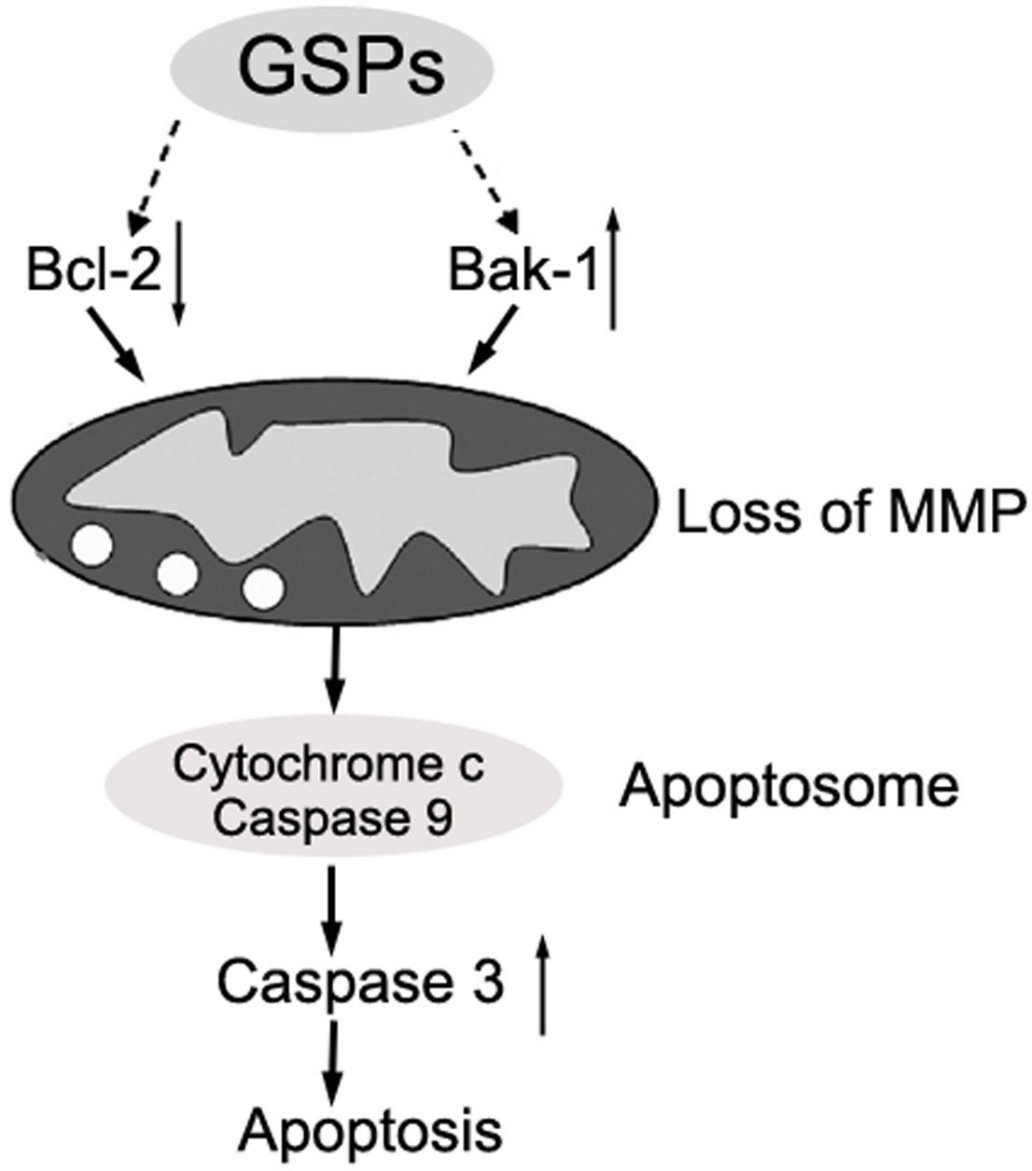
Proposed mechanisms of the GSPs-induced apoptosis of cervical cancer cells. GSPs decrease the expression of Bcl-2 while increasing the expression of Bak-1 and result in the enhancement of mitochondrial membrane permeability associated with the loss of mitochondrial membrane potential (MMP). Subsequently, the release of cytochrome c from mitochondria leads to the activation of caspase-9 and -3, finally resulting in apoptosis.

In summary, our studies demonstrated the anticancer efficacy of GSPs through the induction of apoptosis in cervical cancer cells in vitro and in vivo. Based on our results and other previous research, we speculated that GSPs could trigger the apoptosis of cervical cancer cells through the mitochondrial pathway, characterized by Bcl-2 down-regulation, Bak-1 up-regulation, the loss of mitochondrial membrane potential and the increased activity of caspase-3 (Fig. 9). Therefore, GSPs may be potential bioactive phytochemicals for the chemoprevention or chemotherapy of cervical cancer. However, GSPs are a polyphenolic mixture, and it is therefore necessary to identify the components of GSPs that play key roles in inducing apoptosis. Furthermore, it is still required to elucidate the precise mechanism underlying GSPs-triggered apoptosis; for example, how to inhibit Bcl-2 or/and activate Bak-1 proteins.
